# Diagnostic performance of metagenomic next-generation sequencing in non-tuberculous mycobacterial pulmonary disease when applied to clinical practice

**DOI:** 10.1007/s15010-022-01890-z

**Published:** 2022-08-01

**Authors:** Wei Wei, Jie Cao, Xiao-cui Wu, Li-ping Cheng, Xiao-na Shen, Wei Sha, Qin Sun

**Affiliations:** 1 Shanghai Clinical Research Center for Infectious Disease (Tuberculosis), Shanghai Key Laboratory of Tuberculosis, Shanghai Pulmonary Hospital, School of Medicine, Tongji University, Shanghai, 200433 China; 2 Department of Clinical Laboratory, Shanghai Pulmonary Hospital, School of Medicine, Tongji University, Shanghai, China

**Keywords:** Non-tuberculous mycobacteria, Metagenomic next-generation sequencing, Pulmonary disease, Diagnosis

## Abstract

**Objective:**

To compare non-tuberculous mycobacterial pulmonary disease (NTMPD) diagnosis by metagenomic next-generation sequencing (mNGS) with Bactec mycobacterial growth indicator tube (MGIT) 960.

**Methods:**

A total of 422 patients with suspected NTMPD in Shanghai Pulmonary Hospital between January 2020 and May 2021 were retrospectively analyzed; 194 were diagnosed with NTMPD. The diagnostic performance of mNGS and MGIT 960 for NTMPD was assessed. Receiver operating characteristic (ROC) curves and areas under curve (AUCs) were compared.

**Results:**

The sensitivity of mNGS in NTMPD diagnosis was 81.4% and higher than that of MGIT 960 (53.6%). The specificity of mNGS in NTMPD diagnosis was 97.8%, similar to that of MGIT 960 (100%). The sensitivity of combined mNGS and MGIT 960 in NTMPD diagnosis was 91.8%. The sensitivity of mNGS for bronchoalveolar lavage fluid (BALF), pulmonary puncture tissue fluid, and sputum was 84.8%, 80.6%, and 77.5%, respectively; all were higher than that of MGIT 960 (*P* < 0.05). The AUC of mNGS and MGIT 960 was 0.897 and 0.768, respectively. The AUC of mNGS were BALF (0.916), pulmonary puncture tissue fluid (0.903), and sputum (0.870).

**Conclusion:**

The sensitivity of mNGS was superior to that of Bactec MGIT 960; the specificity in NTMPD diagnosis was similar. mNGS shows effective performance in NTMPD diagnosis.

## Introduction

Non-tuberculous mycobacteria disease is caused by infection with nontuberculous mycobacteria (NTM) pathogens and is accompanied by pathological changes of related tissues and organs [1–3], among which pulmonary infection is the most common [4]. Recent data have shown an increasing incidence and mortality of non-tuberculous mycobacterial pulmonary disease (NTMPD) worldwide, and NTMPD has become one of the important public health problems threatening human health [5–9]. To date, over 190 species and 14 subspecies of NTM have been discovered (http://www.bacterio.net/mycobacterium.html) [10], and the sensitivity to drugs, treatment regimens and prognosis vary greatly among the species [11–15]. The NTM *Mycobacterium abscessus* can be transmitted from person to person, indicating that environmental exposure is no longer the only way for NTM transmission [16]. Therefore, the early diagnosis, timely treatment and appropriate management for NTMPD patients are of great importance.

NTM is morphologically similar to *Mycobacterium tuberculosis* (MTB). NTMPD may cause similar clinical manifestations as pulmonary tuberculosis, resulting in misdiagnosis and ineffective treatments [17]. Acid-fast staining can rapidly detect acid-fast bacilli but cannot distinguish MTB from NTM. The gold standard for NTM diagnosis is the traditional culture method of Bactec mycobacterial growth indicator tube (MGIT) 960, but it is limited by its long detecting cycle (4–8 weeks) and low positive rate; furthermore, it cannot distinguish the specific strains of NTM, which brings challenges for rapid diagnosis and treatment in clinical practice. Currently, matrix-assisted laser desorption ionization time of flight mass spectrometry, 16S rRNA gene sequencing, line probe assay, fluorescence PCR dissolution curve and other technologies have been widely used in clinical applications; these techniques can rapidly detect NTM pathogens and drug sensitivity simultaneously. However, their use is limited to the known NTM strains in the database, and new species of NTM [18–20] and other pathogens cannot be identified using these technologies. Therefore, there is an urgent need for a rapid and accurate method for NTM detection in clinical practice.

Metagenomic next-generation sequencing (mNGS), a new molecular detection method, has a high-throughput and faster than the previously developed strategies and allows for an unbiased approach for the detection of pathogens, which is of value for the diagnosis of infectious diseases [21]. In recent years, mNGS has been widely applied in the identification of infectious pathogens such as bacteria, fungi, and viruses [22–24], showing good performance in clinical application. Studies have found that mNGS was highly valuable in the diagnosis of active MTB complex infection, with the sensitivity of 44–89.13% and the specificity of 98–100% [25–27]. Some studies have found that mNGS can identify NTM and the specific strains [28, 29], but most of these studies were case reports and did not systematically evaluate the diagnostic value of mNGS in clinical practice. Therefore, our study compared mNGS with the traditional culture method of Bactec MGIT 960 to explore the diagnostic performance of mNGS in NTMPD.

## Materials and methods

### Study design, patients and samples

A total of 528 patients with suspected NTMPD in Shanghai Pulmonary Hospital affiliated to Tongji University between January 1, 2020 and May 31, 2021 were enrolled in this retrospective study. Among the 528 patients, 422 patients were included according to the following criteria. The inclusion criteria were as follows: (1) patients aged from 16 to 75 years, no sex limitation; (2) patients with negative HIV result; (3) patients with pulmonary lesions in accordance with the imaging changes of NTMPD, including fibrous cavities, multifocal bronchiectasis, multiple nodules, and mass shadow; and with negative results of acid-fast staining but NTM infection could not be ruled out; (4) patients with one or more positive results in acid-fast staining of smears of sputum or BALF but negative MTB result in molecular biological tests; and (5) patients for which only one positive NTM result in the culture was obtained. Exclusion criteria were as follows: (1) patients with incomplete clinical data; (2) patients with only mNGS performed or only MGIT 960 performed; and (3) patients with unclear diagnosis.

**Fig. 1:**
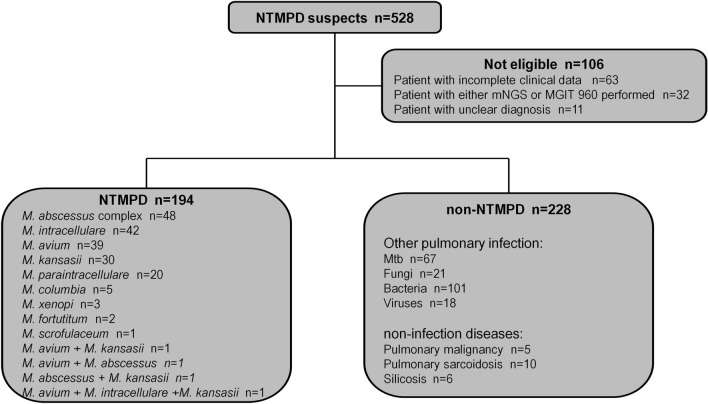
The flow diagram of the study

Among the 422 patients, 194 were in the NTMPD group, including 48 with *M. abscessus* complex, 42 with *Mycobacterium intracellulare*, 39 with *Mycobacterium avium*, 30 with *Mycobacterium kansasii*, 20 with *Mycobacterium paraintracellulare*, 5 with *Mycobacterium columbia*, 3 with *Mycobacterium xenopi*, 2 with *Mycobacterium fortuitum*, and 1 with *Mycobacterium scrofulaceum*. Two or more types of NTM were detected simultaneously in four patients: *M. avium* + *M. kansasii* in 1 patient, *M. avium* + *M. abscessus* in 1 patient, *M. abscessus* + *M. kansasii* in 1 patient, and *M. avium* + *M. intracellulare* + *M. kansasii* in 1 patient. There were 228 patients in the non-NTMPD group, including 207 with other pulmonary infectious diseases (67 infected with *M. tuberculosis*, 21 infected with fungi, 101 infected with bacteria, 18 infected with viruses) and 21 with non-infectious diseases (5 with pulmonary malignancy, 10 with pulmonary sarcoidosis, 6 with silicosis), as shown in Fig. 1. Clinical characteristics such as demographic data, chest computed tomography (CT) imaging, specimen types and laboratory results were recorded in detail for each included patient.

### Diagnostic criteria of NTMPD

According to the guidelines of American Thoracic Society/European Respiratory Society/European Society of Clinical Microbiology and Infectious Diseases/Infectious Diseases Society of America (ATS/ERS/ESCMID/IDSA) (2020) [1] and the Guidelines of Chinese Medical Association for the Diagnosis and Treatment of Non-tuberculous Mycobacteriosis (2020), for patients with pulmonary or systemic symptoms, nodular or cavitary opacities on chest radiograph or a high-resolution CT scan that shows bronchiectasis with multiple small nodules, after appropriate exclusion of other diagnoses and ensuring no exogenous contamination of specimens, patients who meet one of the following conditions can be diagnosed as NTMPD: (1) positive culture results from at least two separate expectorated sputum samples and/or the same results with the molecular biological tests; (2) positive culture results from at least one bronchial wash or BALF; (3) electronic bronchoscope or other lung biopsy with mycobacterial histologic features (granulomatous inflammation or acid-fast bacilli) and positive culture or molecular biological tests for NTM; and (4) electronic bronchoscope or other lung biopsy with mycobacterial histologic features (granulomatous inflammation or acid-fast bacilli) and one or more sputum or BALF samples that were culture positive for NTM.

In this study, the species of *Mycobacterium* in the respiratory samples were identified by one of the following methods: (1) NTM strains positive for MGIT 960 was further compared using 16S DNA to determine the specific species. (2). PCR-reverse dot blot hybridization assay (Yaneng Biotechnology Co., Ltd); 3. Matrix-assisted laser desorption ionization time of flight mass spectrometry (MALDI-TOF MS) (MassARRAY^®^ System). The results of these assays were carefully evaluated to determine whether they were consistent with clinical conditions of the patients.

### mNGS (Beijing Genomics Institute)

#### Specimen collection

Sputum collection was carried out under the guidance of doctors and nurses. Patients were required to collect morning sputum through deeply coughing after gargling with clean water. BALF and pulmonary puncture tissue fluid were collected according to the standard procedures.

Specimen transfer: sputum /BALF/ pulmonary puncture tissue fluid (1.5–3 ml) was collected in a tube without deoxyribonuclease (Dnase), cryopreserved at − 20 ℃, and delivered to the laboratory within 24 h.

#### Specimen pretreatment

The collected BALF and sputum were liquefied for 10-15 min at 37 ℃, and then centrifuged at 12,000×*g* for 5 min. Pathogens were enriched at 37 ℃ for 20 min, and the supernatant (600 ul) was transferred to the tube. Puncture tissue fluid were ground into homogenized tissue, and the supernatant (600 ul) was transferred to the tube.

#### DNA extraction

Lyticase (7.2 µl; RT410-TA, Tiangen Biotech, Beijing, China) was added to the sample. After mixing and shaking, 300 μl samples were taken and DNA was extracted using the TIANamp Micro DNA Kit (DP316, Tiangen Biotech) according to the instructions.

#### Library construction and sequencing

The extracted DNA was subjected to enzyme digestion, terminal repair, connector connection and PCR amplification for library construction. Agilent 2100 Bioanalyzer was applied for quality control to make sure that the size of fragments in the constructed DNA library reached up to 300 bp., and the concentration was measured by the Qubit dsDNA HS Assay Kit (Thermo Fisher Scientific Inc.) as recommended by the manufacturer. DNA pools were constituted with equal amounts of DNA from individual samples. The library after DNA pooling was formed into a long single stranded DNA using a circular DNA template, and DNA Nano Balls (DNBs) were generated through rolling circle amplification (RCA). The prepared DNBs were loaded onto a sequencing chip and sequenced with the BGISEQ-50/MGISEQ-2000. Optical signals were collected using a high-resolution imaging system and then converted into digital information, which was then decoded into DNA sequence information.

#### Data analysis

To control the impact of contamination, negative controls were prepared in parallel and sequenced in the same operation. Clinical specimens contain human nucleic acid and microbial nucleic acid, and all types of nucleic acid in the samples are tested. Low-quality and low-complexity regions were removed to obtain high quality data. Based on BWA (BWA: http://biobwa.sourceforge.net/), high quality data was aligned to the human genome to remove human reads and then aligned with processed nonhuman sequencing reads to a curated pathogen database (including 6350 species of bacteria, 1064 species of fungi, 4945 species of viruses, 234 species of parasites) so as to achieve a taxonomic classification to each sequence read for microbial identification.

### BACTEC MGIT 960

The operating procedure for BACTEC MGIT 960 was as following: (1) The mixture of NaOH (50 mL, 2–4%), sodium citrate (50 ml, 2.9%) as well as N-Acetylcysteine (NAC, 0.5 g) were added into the specimen of sputum (1 ml), followed by the vibration for 15 min; (2) The sterile PBS solution (1/15 m, pH 6.8) was added into the specimen, mixed gently, and then centrifuged at 3000 g for 15 min; (3) The supernatant was the specimen was discarded and the sterile PBS (2 ml) was added into the remaining; (4) The sterile oleic acid-albumin-dextrose-catalase (OADC) / polymyxin-B, amphotericin-B, nalidixic acid, trimethoprim, azlocillin (PANTA) (0.8 ml) was added into the mycobacteria growth indicator tube (MGIT), followed by 0.5 ml pretreated specimen; (5) MGIT was placed in the BACTEC MGIT 960 and incubated for 6 weeks.

The isolates with positive results of MGIT 960 were further verified into MTBC by MPB64 antigen, and tests for the growth media of rho-nitrobenzoic acid (PNB) and Thiophene-2-carboxylic acid hydrazide (TCH) were performed. PNB and TCH were used as the differential media for preliminary identification of the species of mycobacterium, including MTBC, Mycobacterium bovis or NTM. MTBC and Mycobacterium bovis could not grow on the medium containing 0.5 mg/ml PNB, and Mycobacterium bovis could not grow on the medium containing 5 mg/ml TCH, but majority of NTMs could grow on both of the two media. Thus, the preliminary results obtained from the differential media were as followings: (1) MTBC: TCH ( +) and PNB (–); (2) *Mycobacterium* bovis: TCH (–) and PNB (–); (3) NTM: TCH ( +) and PNB ( +).

### Statistical analysis

SPSS26.0 software (SPSS Inc., Chicago, IL, USA) was used for statistical analysis. Continuous variables with normal distribution were expressed as mean ± standard deviation (SD). Student’s *T* test was used for comparison of the means, and *χ*^2^ test was used for the comparison of the categorical data between groups. The diagnostic performance of mNGS as well as MGIT 960 for NTMPD was calculated, including the sensitivity, specificity, positive predictive value (PPV), positive likelihood ratio (PLR), negative likelihood ratio (NLR), and Youden's index. Besides, the receiver operating characteristic (ROC) curve was drawn and the area under the curve (AUC) was calculated. McNemar test was used to compare the sensitivity of the two techniques. All tests were two-sided, and *P* < 0.05 was considered to be statistically significant.

## Results

### Baseline comparison of demographic and clinical characteristics between the NTMPD and non-NTMPD groups

Patient characteristics are listed in Table 1. There were 81 males (42.1%) and 113 females (57.9%) in the NTMPD group, and the average age was 55.6 ± 13.7 years. There were 111 males (48.6%) and 117 females (51.4%) in the non-NTMPD group, and the average age was 55.4 ± 13 years. There were no significant differences in sex, age, body mass index, imaging findings and other baseline characteristics between the two groups. In the NTMPD group, 71 (36.1%) sputum, 92 (57.2%) BALF, and 31 (6.7%) pulmonary puncture fluid samples were collected. In the non-NTMPD group, 87 (38.1%) sputum, 131 (57.5%) BALF, and 10 (4.4%) pulmonary puncture fluid samples were collected.

**Table 1:** Comparison of baseline demographic and clinical characteristics between the NTMPD and non-NTMPD groups

	NTMPD group *n* = 194	Non-NTMPD group *n* = 228	*P* value
Age (year)	55.6 ± 13.7	55.4 ± 13	> 0.05
Sex (*n*, %)			
Female	113(57.9%)	117(51.4%)	> 0.05
Male	81(42.1%)	111(48.6%)	> 0.05
BMI (kg/m^2^)	19.2 ± 7	20.7 ± 6.8	> 0.05
Radiological characteristics			
Bronchiectasis	84.5%(164/194)	77.6%(177/228)	> 0.05
Nodular opacities	75.7%(147/194)	72%(164/228)	> 0.05
Cavitary opacities	68%(132/194)	57%(130/228)	> 0.05
Specimen (*n*, %)			
Sputum	71(36.6%)	87(38.1%)	> 0.05
BALF	92(47.4%)	131(57.5%)	> 0.05
Pulmonary puncture fluid	31(16%)	10(4.4%)	> 0.05

*NTMPD* non-tuberculous mycobacterial pulmonary disease, *BMI* body mass index, *BALF* bronchoalveolar lavage fluid

### Consistency between mNGS and MGIT 960

**Fig. 2:**
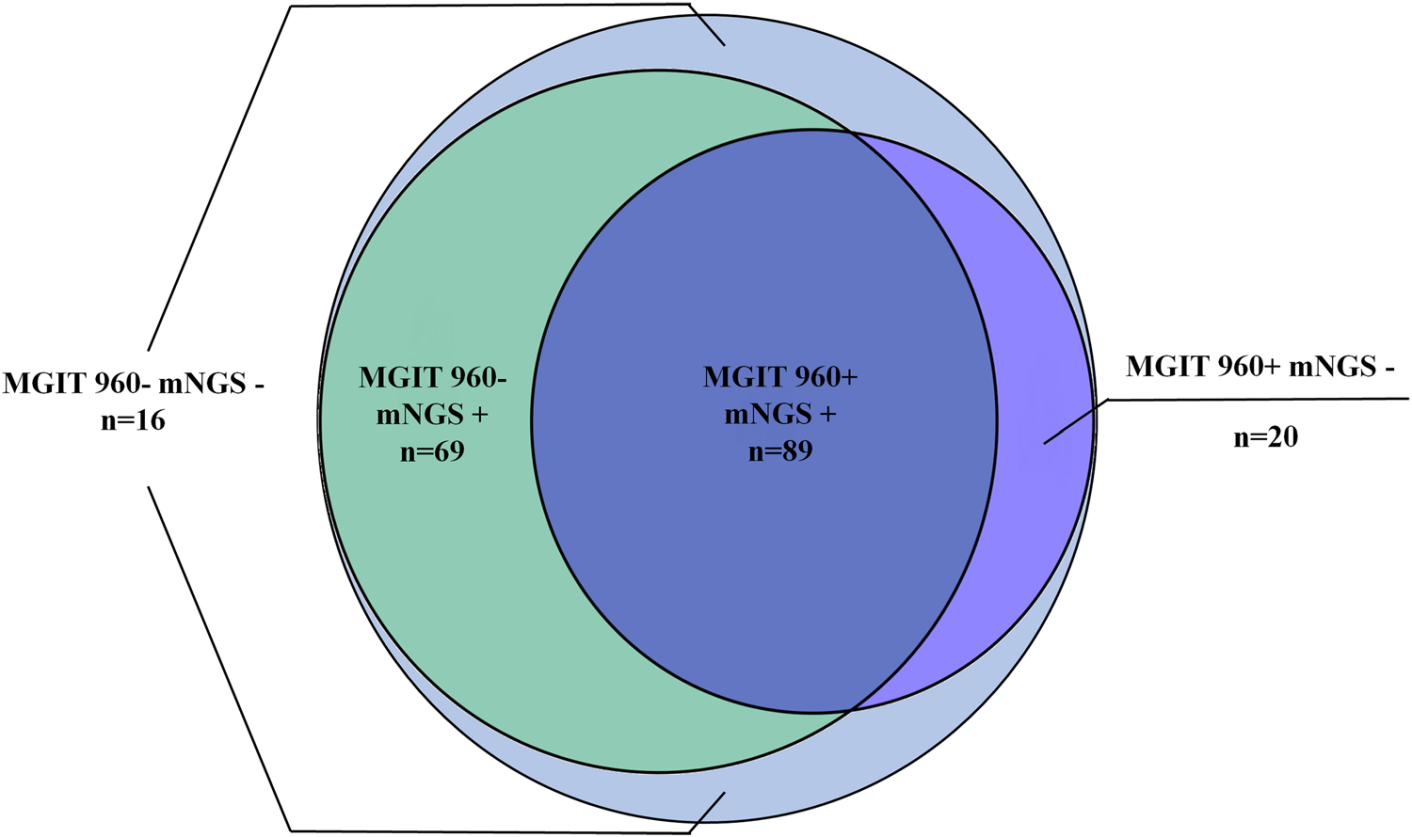
Consistency of the results for NTM between mNGS and MGIT 960

Among the 194 patients with NTMPD, 89 were positive for NTM in both mNGS and MGIT 960, and 16 were negative in both mNGS and MGIT 960; 69 patients were only positive in mNGS, and 20 patients were only positive in MGIT 960 (Fig. 2). Notably, in five patients, mNGS detected positive NTM while MGIT 960 reported negative results. After evaluation of clinical manifestation and the images, the positive NTM results from mNGS in the five patients were considered as a false positive.

### Diagnostic performance of mNGS and MGIT 960 alone and in combination for NTMPD

The median of the number of NTM-specific sequences detected by mNGS in 194 patients with NTMPD was 37 (range 4–1576; inter-quartile ranges [IQR] 24–89); the median of the coverage of NTM genome detected by mNGS was 0.0182 (range 0.0017–0.1464, IQR 0.0073–0.0627). The total sensitivity of mNGS (81.4% [158/194]) in all clinical specimens was much higher than that of MGIT 960 (53.6% [104/194]). The total specificity of mNGS (97.8% [223/228]) in all clinical specimens was slightly lower than that of MGIT 960 (100% [194/194]).

The sensitivity of mNGS in different specimens ranked as follows: BALF (84.8% [78/92]) > pulmonary puncture tissue fluid (80.6% [25/31]) > sputum (77.5% [55/71]). The specificity of mNGS was as follows: BALF, 98.4% (129/131); pulmonary puncture fluid, 100% (31/31); and sputum, 96.5% (84/87). The sensitivity of MGIT 960 in different specimens ranked as follows: BALF (60.9% [56/92]) > pulmonary puncture fluid (58.1% [18/31]) > sputum (49.3% [35/71]).

The sensitivity of the combination of mNGS and MGIT 960 was greatly improved to 91.8%, which was higher than that of mNGS or MGIT 960 alone (81.4 and 53.6%). The sensitivity of the combination of mNGS and MGIT 960 in the sputum, BALF, and pulmonary puncture fluid was also increased, reaching up to 93.0, 91.3 and 90.3%, respectively, as shown in Table 2.

**Table 2:** Diagnostic performance of mNGS and MGIT 960 for NTMPD

Clinical specimen		Sensitivity% (*n*/*N*)	Specificity% (*n*/*N*)	PPV%	NPV%	PLR	NLR	YD
Total								
mNGS		81.4% (158/194)	97.8% (223/228)	96%	86.1	37	0.19	0.792
MGIT 960		53.6% (104/194)	100% (228/228)	100	71.6	0	0.464	0.536
Combination of mNGS and MGIT 960		91.8% (178/194)	100% (228/228)	98.9	94.2	0	0.082	0.909
Sputum								
mNGS		77.5% (55/71)	96.5% (84/87)	94.8	84	22.14	0.23	0.74
MGIT 960		49.3% (35/71)	100 (87/87)	100	70.7	0	0.507	0.493
Combination of mNGS and MGIT 960		93.0% (66/71)	100% (87/87)	100	93.5	0	0.07	0.930
BALF								
mNGS		84.8% (78/92)	98.4% (129/131)	97.5	90.2	53	0.154	0.832
MGIT 960		60.9% (56/92)	100 (131/131)	100	78.4	0	0.391	0.609
Combination of mNGS and MGIT 960		91.3%(84/92)	100%(131/131)	98.1	94.2	0	0.087	0.898
Pulmonary puncture fluid								
mNGS	80.6% (25/31)		100% (10/10)	100	62.5	0	0.194	0.806
MGIT 960	58.1% (18/31)		100% (10/10)	100	43.4	0	0.419	0.581
Combination of mNGS and MGIT 960	90.3% (28/31)		100% (10/10)	100	75.2	0	0.097	0.903

*mNGS* metagenomic next-generation sequencing, *MGIT* mycobacterial growth indicator tube, *BALF* bronchoalveolar lavage fluid, *PPV* positive predictive value, *NPV* negative predictive value, *PLR* positive likelihood ratio, *NLR* negative likelihood ratio, *YD* Youden index

### Comparison of the diagnostic performance between mNGS and MGIT 960

To compare the difference in the sensitivity between mNGS and MGIT 960, we performed the McNemar test and found that mNGS had a higher sensitivity than MGIT 960 in 194 specimens from NTMPD patients (83.5% vs 57.2%, *P* < 0.001). The sensitivity of mNGS in the sputum was significantly higher than that of MGIT 960 (77.5% vs 49.3%; *P* = 0.003); the sensitivity of mNGS in BALF was significantly higher than that in MGIT 960 (84.8 vs 60.9%; *P* < 0.001). Although the sensitivity of mNGS in pulmonary puncture fluid tended to be higher than that of MGIT 960 (80.6 vs 50.1%), the difference was not statistically significant (*P* = 0.092).

### ROC curves of mNGS and MGIT 960

**Fig. 3:**
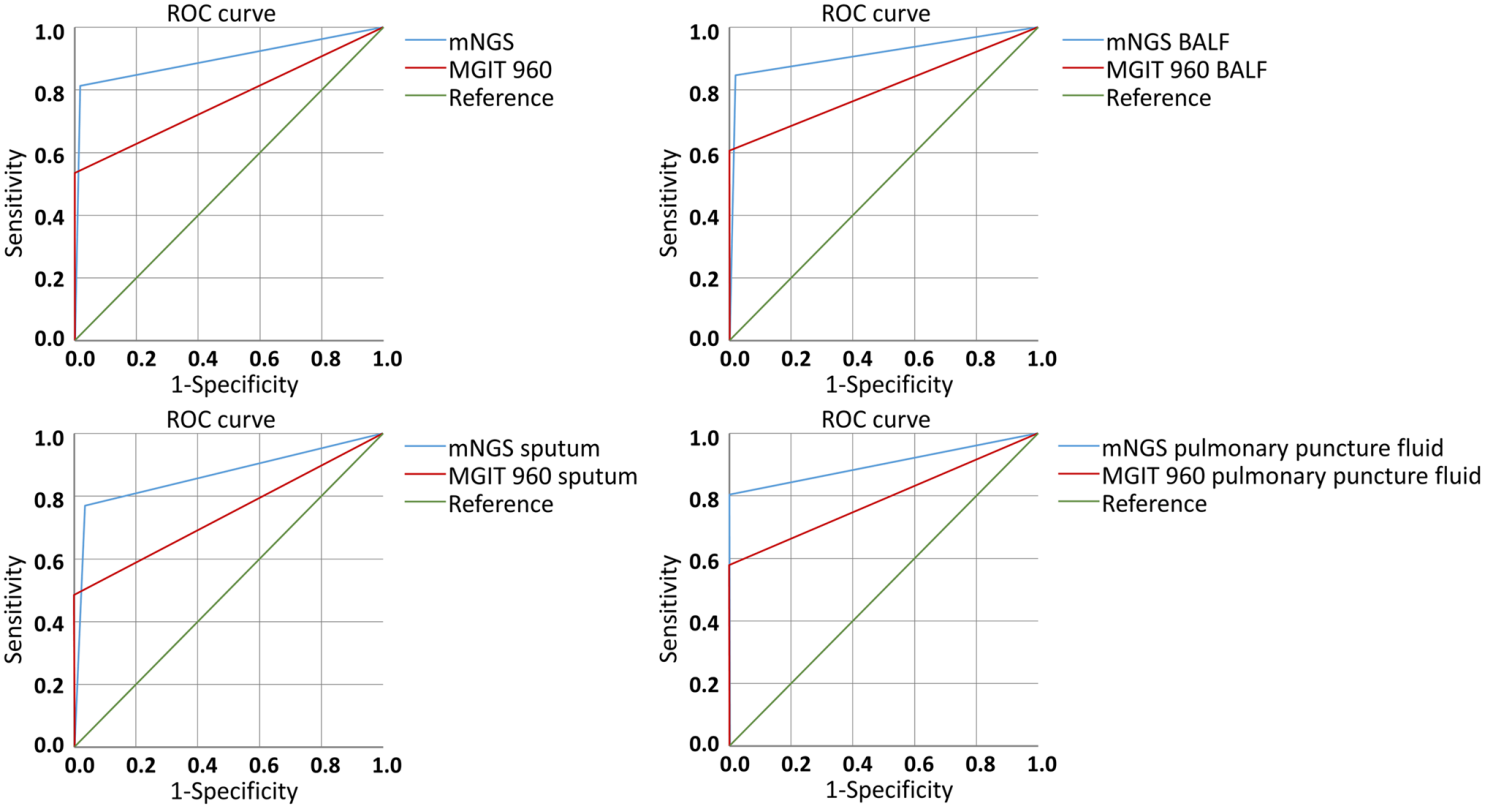
ROC curves of mNGS and MGIT 960

The AUC of mNGS was 0.897 (95% CI 0.863–0.932), which was higher than that of MGIT 960 (0.768 [95% nCI 0.720–0.816]). The AUCs of mNGS for different specimens ranked as follows: BALF (0.916 [95%CI 0.871–0.962]) > pulmonary puncture fluid (0.903 [95% CI 0.812–0.995]) > sputum (0.870 [95% CI 0.807–0.933]). The AUC of MGIT 960 for different specimens ranked as follows: BALF (0.804 [95% CI 0.739–0.869]) > pulmonary puncture fluid (0.790 [95% CI 0.655–0.925]) > sputum (0.746 [95% CI 0.655–0.828]), as shown in Fig. 3.

## Discussion

Although the diagnostic performance of mNGS in patients with different infections has been examined [22–27], reports on its diagnostic value in NTMPD patients are rare. In this study, the diagnostic performance of mNGS and MGIT 960 for NTMPD was comprehensively and systematically compared through a single-center retrospective study based on a relatively large sample size (422 patients with suspected NTM, including 194 patients with NTMPD), and the diagnostic efficacy of mNGS in different clinical specimens (BALF, pulmonary puncture fluid, and sputum) was also compared. Our results showed that the total sensitivity of mNGS was 81.4%, and the sensitivity of mNGS in BALF was the highest (84.8%) compared with that of other specimen types and much higher than that reported in previous studies. A retrospective study of 347 patients with infectious diseases by Miao et al. reported that the sensitivity of mNGS for NTM was only 29.8% [30]. Shi et al. investigated the performance of mNGS on BALF samples from 110 suspects with pulmonary tuberculosis and found that mNGS identified 63.63% cases of NTM [31]. We speculate that the underlying reason for the difference of the sensitivities of mNGS in the different studies was because of the inclusion criteria. The patients included in our study were patients suspected for NTMPD with suspected lesions in the lung; some patients had already shown positive results of acid-fast staining in sputum smears, and a small number of patients presented one positive NTM result in the culture. In contrast, the patients included in previous studies were those with infectious diseases or TB suspects. This may explain the higher sensitivity of mNGS in the diagnosis of NTMPD in this study than that in other previous studies. The sensitivity of the combination of mNGS and MGIT 960 was as high as 91.8%, and we suggest the combination be used for patients with suspected NTMPD in clinical practice to increase the detection rate of NTM and facilitate the diagnosis of NTMPD.

This study also compared the diagnostic performance of mNGS and MGIT 960 in different clinical specimens of NTMPD. Our results showed that the sensitivity of mNGS in BALF, pulmonary puncture fluid and sputum was 84.8, 80.6, and 77.5%, respectively, while the sensitivity of MGIT 960 in BALF, pulmonary puncture fluid and sputum was 60.9, 58.1, and 49.3%, respectively, which was consistent with the results reported by Miao et al. [30]. Although MGIT 960 is the current gold standard for the diagnosis of NTM, its sensitivity is significantly lower than mNGS. The possible reasons are as follows: (1) Some strains of NTM are difficult to grow in the culture, and some strains of NTM need special medium, specific temperature, or the prolonged time for culture. Few biological laboratories could meet with these requirements and easy to obtain false negative results; (2) Improper collection or handling of specimens may cause the contamination, especially in the collection of sputum, resulting in the failure or false negative results of the culture; (3) compared to the strain of NTM, DNA in mNGS could survive longer in the collected specimens and was more convenient for detection, making the diagnostic sensitivity of mNGS higher than that of MGIT 960. Therefore, we recommend bronchoscopy to collect BALF from patients with suspected NTMPD for mNGS to improve diagnostic sensitivity. For patients with mass or nodular lesions, CT-guided percutaneous puncture of the lung was a good choice for specimen collection.

Another advantage of mNGS is the simultaneous and independent sequencing of massive amounts of DNA without requirement for prior knowledge. A significant proportion of NTMPD patients have underlying pulmonary disease (e.g., bronchiectasis, COPD) and these patients may have multiple infections, which were difficult to detect by conventional culture method or molecular detection technology for a single pathogen, resulting in misdiagnosis or missed diagnosis. In the specimens, only the sputum could be reproducibly detected. Considering the difficulty of specimen collection through invasive examinations, such as bronchoscopy and punctures, the establishment of one method that allows for the detection of pathogens using one specimen to guide clinical diagnosis and treatment is advantageous. In our NTMPD group, mNGS detected 7 patients with co-infection, including tuberculosis, *Pseudomonas aeruginosa*, *Candida albicans*, *Aspergillus*, *Cryptococcus*, and virus, indicating that mNGS had obvious advantages over other molecular detection techniques in the diagnosis of NTMPD accompanied with co-infections of other pathogens. Additionally, mNGS has a shorter detection cycle, which enables clinicians to obtain etiological evidence within 3 days compared with the average feedback time of MGIT 960 ranging from 30 to 45 days. Thus, mNGS has been recognized as an advantageous method for the early and rapid diagnosis of diseases in clinical practice.

In five patients in the study, mNGS detected positive NTM while MGIT 960 showed negative results. After evaluation of clinical manifestations and the images, the positive NTM results were determined as false positive in all five patients. Three sources may explain the false positive results. One source may be from the mNGS procedures, including contaminant pathogen DNA across samples during mNGS library preparation, low-complexity sequences matching low-quality reads from the sample, misannotated species, or contaminants from database entries that also contain reads to human DNA, sequencing adaptors, or vector colonization. The second type of false positive may be from the process before mNGS procedures, mainly from contamination acquired throughout sample collection and processing, which can only be excluded by clinical diagnosis or resampling if possible [25]. The third potential source for the false positive result is from the limitations of mNGS, which are similar with those other molecular technologies, as these techniques cannot determine whether the detected NTM is a colonized, background or pathogenic bacteria or if it is alive or dead. This information can only be resolved through a comprehensive assessment based on clinical manifestations and results of other examinations.

This study has several limitations. Although the study included a relatively large sample size, it was a single-center retrospective study. The diagnostic performance of mNGS for NTMPD still needs to be verified by multi-center and prospective studies. In addition, whether mNGS has potential in improving the prognosis of NTMPD patients through early diagnosis still needs to be studied. Finally, although mNGS is expensive, its cost-effectiveness needs to be further addressed.

## Conclusion

Compared with the traditional culture method of MGIT 960, mNGS showed a better sensitivity but a slightly lower specificity for the diagnosis of NTMPD because of its low false positivity, indicating that its potential for clinical application. Additionally, the combination of mNGS and MGIT 960 presented even higher sensitivity than either system alone. In terms of the clinical specimens, BALF and pulmonary puncture fluid showed better performance than sputum in evaluation by mNGS. Furthermore, mNGS was able to detect NTMPD co-infected with other pathogens. In conclusion, our results indicate that mNGS is a rapid and effective method for the diagnosis of NTMPD in clinical practice.

## Data Availability

The datasets used and/or analyzed during the current study are available from the corresponding author on reasonable request.

## References

[1] Daley CL, Iaccarino JM, Lange C (2020). Treatment of nontuberculous mycobacterial pulmonary disease: an official ATS/ERS/ESCMID/IDSA clinical practice guideline. Eur Respir J.

[2] Haworth CS, Banks J, Capstick T (2017). British thoracic society guidelines for the management of non-tuberculous mycobacterial pulmonary disease (NTM-PD). Thorax.

[3] Gopalaswamy R, Shanmugam S, Mondal R (2020). Of tuberculosis and non-tuberculous mycobacterial infections—a comparative analysis of epidemiology, diagnosis and treatment. J Biomed Sci.

[4] Porvaznik I, Solovič I, Mokrý J (2017). Non-tuberculous mycobacteria: classification, diagnostics, and therapy. Adv Exp Med Biol.

[5] Furuuchi K, Morimoto K, Yoshiyama T (2019). Interrelational changes in the epidemiology and clinical features of nontuberculous mycobacterial pulmonary disease and tuberculosis in a referral hospital in Japan. Respir Med.

[6] Prevots DR, Marras TK (2015). Epidemiology of human pulmonary infection with nontuberculous mycobacteria: a review. Clin Chest Med.

[7] Smith GS, Ghio AJ, Stout JE (2016). Epidemiology of nontuberculous mycobacteria isolations among central North Carolina residents, 2006–2010. J Infect.

[8] Morimoto K, Iwai K, Uchimura K (2014). A steady increase in nontuberculous mycobacteriosis mortality and estimated prevalence in Japan. Ann Am Thorac Soc.

[9] Vinnard C, Longworth S, Mezochow A (2016). Deaths related to nontuberculous mycobacterial infections in the united states, 1999–2014. Ann Am Thorac Soc.

[10] Euzéby JP (1997). List of bacterial names with standing in nomenclature: a folder available on the internet. Int J Syst Bacteriol.

[11] Ratnatunga CN, Lutzky VP, Kupz A (2020). The rise of non-tuberculosis mycobacterial lung disease. Front Immunol.

[12] Koh WJ, Jeong BH, Kim SY (2017). Mycobacterial characteristics and treatment outcomes in *Mycobacterium*
*abscessus* lung disease. Clin Infect Dis.

[13] Chapagain M, Gumbo T, Heysell SK (2020). Comparison of a novel regimen of rifapentine, tedizolid, and minocycline with standard regimens for treatment of pulmonary *Mycobacterium* kansasii. Antimicrob Agents Chemother.

[14] Griffith DE, Eagle G, Thomson R (2018). Amikacin liposome inhalation suspension for treatment-refractory lung disease caused by *Mycobacterium* avium complex (convert). A prospective, open-label, randomized study. Am J Respir Crit Care Med.

[15] Cheng LP, Chen SH, Lou H (2022). Factors associated with treatment outcome in patients with nontuberculous mycobacterial pulmonary disease: a large population-based retrospective cohort study in shanghai. Trop Med Infect Dis.

[16] Bryant JM, Grogono DM, Greaves D (2013). Whole-genome sequencing to identify transmission of *Mycobacterium abscessus* between patients with cystic fibrosis: a retrospective cohort study. Lancet.

[17] Raju RM, Raju SM, Zhao YL (2016). Leveraging advances in tuberculosis diagnosis and treatment to address nontuberculous mycobacterial disease. Emerg Infect Dis.

[18] Toney NC, Toney SR, Butler WR (2010). Utility of high-performance liquid chromatography analysis of mycolic acids and partial 16S rRNA gene sequencing for routine identification of *Mycobacterium* spp. in a national reference laboratory. Diagn Microbiol Infect Dis.

[19] Ceyssens P, Soetaert K, Timke M (2017). Matrix-assisted laser desorption ionization-time of flight mass spectrometry for combined species identification and drug sensitivity testing in mycobacteria. J Clin Microbiol.

[20] Xu Y, Liang B, Du C (2019). Rapid identification of clinically relevant *Mycobacterium* species by multicolor melting curve analysis. J Clin Microbiol.

[21] Gu W, Miller S, Chiu CY (2019). Clinical metagenomic next-generation sequencing for pathogen detection. Annu Rev Pathol.

[22] Wilson MR, Naccache SN, Samayoa E (2014). Actionable diagnosis of neuroleptospirosis by next-generation sequencing. N Engl J Med.

[23] Langelier C, Zinter MS, Kalantar K (2018). Metagenomic sequencing detects respiratory pathogens in hematopoietic cellular transplant patients. Am J Respir Crit Care Med.

[24] Tang WJ, Zhang Y, Luo C (2021). Clinical application of metagenomic next-generation sequencing for suspected infections in patients with primary immunodeficiency disease. Front Immunol.

[25] Zhou X, Wu HL, Ruan QL (2019). Clinical evaluation of diagnosis efficacy of active *Mycobacterium* tuberculosis complex infection via metagenomic next-generation sequencing of direct clinical samples. Front Cell Infect Microbiol.

[26] Liu X, Chen YL, Ouyang H (2021). Tuberculosis diagnosis by metagenomic next-generation sequencing on bronchoalveolar lavage fluid: a cross-sectional analysis. Int J Infect Dis.

[27] Zhu N, Zhou DB, Sq Li (2021). Diagnostic accuracy of metagenomic next-generation sequencing in sputum-scarce or smear-negative cases WITH suspected pulmonary tuberculosis. Biomed Res Int.

[28] Zhu H, Zhu M, Lei JH (2021). Metagenomic next-generation sequencing can clinch diagnosis of non-tuberculous mycobacterial infections: a case report. Front Med (Lausanne).

[29] Xie D, Xian Y, You JY (2021). Co-infection pneumonia with *Mycobacterium*
*abscessus* and Pneumocystis jiroveci in a patient without HIV infection diagnosed by metagenomic next-generation sequencing. Infect Drug Resist.

[30] Miao Q, Ma YY, Wang QQ (2018). Microbiological diagnostic performance of metagenomic next-generation sequencing when applied to clinical practice. Clin Infect Dis.

[31] Shi CL, Han P, Tang PJ (2020). Clinical metagenomic sequencing for diagnosis of pulmonary tuberculosis. J Infect.

